# Effectiveness of Standard Local Anesthetic Bupivacaine and Liposomal Bupivacaine for Postoperative Pain Control in Patients Undergoing Truncal Incisions: A Randomized Clinical Trial

**DOI:** 10.1001/jamanetworkopen.2021.0753

**Published:** 2021-03-16

**Authors:** Harleen K. Sandhu, Charles C. Miller, Akiko Tanaka, Anthony L. Estrera, Kristofer M. Charlton-Ouw

**Affiliations:** 1 McGovern Medical School at the University of Texas Health Science Center at Houston; 2 Memorial Hermann Hospital, Texas Medical Center, Houston; 3 HCA Houston Healthcare, Gulf Coast Division, Houston, Texas; 4 Department of Clinical Sciences, University of Houston College of Medicine, Houston, Texas

## Abstract

**Importance:**

Liposomal bupivacaine for pain relief is purported to last 3 days compared with 8 hours with standard bupivacaine. However, its effectiveness is unknown in truncal incisions for cardiothoracic or vascular operations.

**Objective:**

To compare the effectiveness of single-administration standard bupivacaine vs liposomal bupivacaine in patients undergoing truncal incisions.

**Design, Setting, and Participants:**

This randomized clinical trial enrolled patients undergoing sternotomy, thoracotomy, minithoracotomy, and laparotomy from a single cardiovascular surgery department in an academic medical center between November 2012 and June 2018. The study was powered to detect a Cohen effect size of 0.35 with a power of greater than 80%. Data analysis was performed from July to December 2018.

**Intervention:**

Patients were randomized to standard bupivacaine or liposomal bupivacaine.

**Main Outcomes and Measures:**

Pain was assessed over 3 postoperative days by the Numeric Rating Scale (NRS). Adjunctive opioids were converted to morphine equivalents units (MEU). NRS scores were compared using Wilcoxon rank-sum (3-day area under the curve) and 2-way nonparametric mixed models (daily scale score) to assess time-by-group interaction. Secondary outcomes included cumulative opioid consumption.

**Results:**

A total of 280 patients were analyzed, with 140 in each group (single-administration standard bupivacaine vs liposomal bupivacaine). Mean (SD) age was 60.2 (14.4) years, and 101 of 280 patients (36%) were women. Irrespective of treatment assignment, pain decreased by a mean of approximately 1 point per day over 3 days (β = −0.87; SE = 0.11; mixed model regression *P* < .001). Incision type was associated with pain with patients undergoing thoracotomy (including minithoracotomy) reporting highest median (interquartile range [IQR]) pain scores on postoperative days 1 (liposomal vs standard bupivacaine, 6 [4-8] vs 5 [3-7]; *P* = .049, Wilcoxon rank-sum) and 2 (liposomal vs standard bupivacaine, 5 [4-7] vs 4 [2-6]; *P* = .003, Wilcoxon rank-sum) but not day 3 (liposomal vs standard bupivacaine, 3 [2-6] vs 3 [1-5]; *P* = .10, Wilcoxon rank-sum), irrespective of treatment group. Median (IQR) 3-day cumulative NRS was 12.0 (8.0-16.5) for bupivacaine and 13.5 (9.0-17.0) for liposomal bupivacaine (*P* = .15, Wilcoxon rank-sum) Furthermore, use of opioids was greater following liposomal bupivacaine compared with standard bupivacaine (median [IQR], 41.5 [21.3-73.8] MEU vs 33.0 [17.8-62.5] MEU; *P* = .03, Wilcoxon rank-sum). On multivariable analysis, no interaction by incision type was observed for mean pain scores or opioid use.

**Conclusions and Relevance:**

In this randomized clinical trial involving truncal incisions for cardiovascular procedures, liposomal bupivacaine did not provide improved pain control and did not reduce adjunctive opioid use compared with conventional bupivacaine formulation over 3 postoperative days.

**Trial Registration:**

ClinicalTrials.gov Identifier: NCT02111746

## Key Points

**Question:**

Does liposomal bupivacaine reduce postoperative pain and supplemental opioid use more than standard bupivacaine in cardiothoracic and vascular surgical patients?

**Findings:**

In this randomized clinical trial including 280 participants, no significant difference in pain control was observed between liposomal vs standard formulation bupivacaine.

**Meaning:**

These results do not support superior performance of liposomal bupivacaine compared with bupivacaine for postoperative pain control in cardiothoracic and vascular truncal incisions.

## Teaser

This randomized clinical trial investigates the effectiveness of liposomal bupivacaine vs standard bupivacaine for postoperative pain in patients with truncal incisions for cardiothoracic or vascular procedures.

## Introduction

More than 80% of patients undergoing surgical procedures report acute postoperative pain, with less than half achieving adequate postoperative pain control, and nearly 75% of those reporting the severity as moderate, severe, or extreme.^1,2,3,4^ This is especially true in open heart, aortic, and lung surgical procedures, where painful truncal incisions are required. Adequate postoperative pain management improves the functional recovery and healing period but also contributes to reduction in postsurgical complication risk and faster patient mobilization, thereby reducing the hospital length of stay and health care costs.^5,6^

Short duration of action is a common drawback of most perioperative pain management regimens, including local anesthetic infiltrations lasting for less than 8 hours.^6,7^ An injectable extended-release bupivacaine formulation lasting up to 72 hours has gained popularity. Several studies^8,9,10,11,12^ on various surgical procedures, including hemorrhoidectomy, bunionectomy, mastectomy, and orthopedic surgery, reported a reduction in postoperative pain (up to 30%) and opioid use following intraoperative use of liposomal bupivacaine compared with placebo and active control. One study^13^ integrated the data from 10 randomized, double-blind studies using liposomal bupivacaine via local wound infiltration to assess the efficacy in postoperative pain control and demonstrated substantially prolonged reduction of postsurgical pain, with a greater proportion of patients avoiding use of opioid rescue medication and a lower total opioid consumption over 72 hours in 5 surgical models. A more recent trial^14^ showed no difference in opioid use within 48 hours after laparotomy for gynecologic surgery.

Few studies analyze liposomal bupivacaine efficacy in postoperative pain management for major truncal procedures, including vascular, cardiac, laparotomy, and/or thoracic surgical wounds. One trial^15^ evaluated parasternal nerve blockade and found minimal differences between liposomal bupivacaine vs saline. Most studies using long-acting local anesthesia were done for smaller incisions that did not penetrate the chest or abdominal cavities. We conducted a masked, randomized clinical trial to evaluate the effectiveness of liposomal bupivacaine for postoperative pain control following truncal incisions.

## Methods

### Study Design

**Figure 1.**
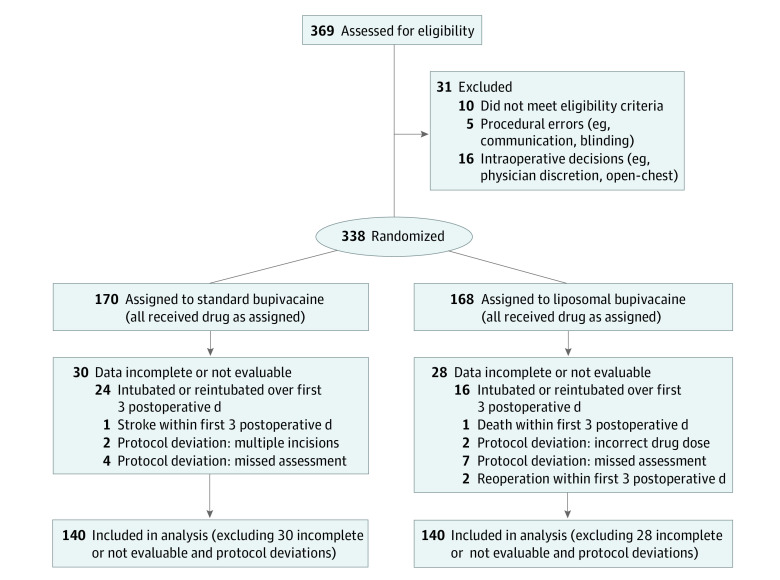
CONSORT Diagram of Participant Flow Through Study

The study was designed as a randomized, masked, active-controlled, parallel-group clinical trial performed at a single institution between November 2012 and June 2018. The study was approved by the University of Texas Health Science Center at Houston Committee for the Protection of Human Subjects. The study conduct and safety was monitored by an independent data safety monitoring board, composed of 2 surgeons with clinical research master’s degrees, an anesthesiologist, and a chaplain, that met periodically during the course of the trial. This study followed the Consolidated Standards of Reporting Trials (CONSORT) reporting guideline (Figure 1).^16^ The trial protocol is shown in Supplement 1.

Because both drug formulations are labeled for use in surgical wound pain control and are in common use for this indication, this was considered to be a comparative effectiveness study, and no support from industry was sought or obtained. Treatment allocation was masked to the patient, the postoperative nursing staff, and the research coordinator conducting the pain and quality of life assessments. Because the appearance of the study drug is different between the liposomal and standard formulations, we did not attempt to mask the treatment group to the surgeon administering the treatment. Most often this was a fellow who had been specifically trained to infiltrate the treatment in a standardized fashion, rather than the attending surgeon. Two separate study coordinators were involved in each case: an unmasked coordinator who obtained the randomization code, consulted with the treating physician, and arranged for the order from the pharmacy, and a masked coordinator who saw the patient daily after surgery and made the postoperative pain scale assessments. Supplemental opioid use was abstracted from the electronic medical record by research personnel masked to group assignment and included all supplemental analgesics delivered by patient-controlled analgesia pump, parenteral injection, or oral route of administration.

### Eligibility and Enrollment

Patients aged 18 years or older who required surgery involving 1 of 4 eligible incisions (median sternotomy, laparotomy, thoracotomy, or minithoracotomy) were eligible to participate. Patients were excluded if they had known allergy to bupivacaine or any opioid, or had long-term opioid exposure or a chronic pain disorder that would make them difficult to evaluate for effectiveness of pain control. Conditions that conferred high probability of postoperative morbidity that could interfere with communication of pain status, such as expectation of intubation for more than 24 hours or altered mental status, were also exclusionary. Signed triplicate consent documents were obtained preoperatively, and adequate time was given to allow for patient and family deliberation. Original documents were included in the physical paper record during the admission. Active participation (assessment of pain and opioid use) was continued for 3 postoperative days. Complication occurrence was monitored for the entire period of hospitalization.

### Administration of Study Drug

Surgery was performed according to routine practice in our group, and no alterations other than treatment with the study drug were made. The 2 treatments were the standard form of bupivacaine hydrochloride (HCl) suspension and a liposomal bupivacaine suspension. Patients in both groups received the same injected volume, 80 mL, divided into 4 20-mL syringes using 22-gauge needles. The liposomal bupivacaine group received a total dose of 266 mg prepared as one 1.3% 20-mL vial of liposomal bupivacaine diluted in 60 mL of preservative-free normal (0.9%) sterile saline for a total volume of 80 mL. The nonliposomal bupivacaine group received a total dose of 125 mg of bupivacaine HCl prepared as one 0.25% 50 mL or five 0.25% 10-mL vials, diluted in 30 mL of preservative-free normal (0.9%) sterile saline for a total volume of 80 mL. At the time of wound closure, the assigned treatment was infiltrated by injection into the tissue surrounding the wound.

### Statistical Analysis

Sample size determination was based on a Cohen effect size of 0.35, which is considered the lower end of the medium effect size range, and for the primary end point would translate to a between-treatment difference of roughly 2.5 scale points for the area under the curve (AUC).^17^ A previous randomized, placebo-controlled trial^12^ for pain management following hemorrhoidectomy demonstrated a Cohen effect size of 0.54, considered a large-medium effect, so we used a smaller hypothesized effect size for our active-controlled trial to ensure adequate power.^17^ We planned 2 interim analyses using the α spending function of O’Brien and Fleming^18^ and determined that a total sample size of 280 evaluable participants would require a final α of 0.0462 to return β = 0.17. The study was not powered to identify differences between incision types, although the randomization was stratified by incision type to ensure balanced treatment allocation within each incision. The randomization schedule was developed using a computer program in blocks of 4 to 6.

Stopping rules were prespecified, with *P* < .0002 required at the first interim analysis and *P* < .012 at the second to stop for efficacy. Sample size was calculated using PASS statistical software version 13 (NCSS, LLC). Stopping for safety, had it been necessary, would have been a determination made by the data safety monitoring board.

The primary end point was incisional pain over the first 3 postoperative days as recorded on the Numeric Rating Scale (NRS), an 11-point ordinal scale ranging from 0 (no pain) to 10 (worst pain imaginable).^19,20,21,22^ We considered an NRS score greater than 4 as poorly controlled pain and a change in 2 points to be clinically meaningful.^23^ Participants were asked to self-aggregate their pain during the previous 24 hours using the NRS. Secondary end points were scores on the Brief Pain Inventory (BPI),^24,25^ patient satisfaction with postoperative pain ratings (using a 5-point Likert scale, where 1 = extremely dissatisfied, 2 = somewhat dissatisfied, 3 = neutral, 4 = somewhat satisfied, and 5 = extremely satisfied), and cumulative opioid analgesic consumption over the first 3 postoperative days. We also evaluated postoperative length of stay, postoperative complications, and mortality. The pain scales are all ordinal and so were compared in univariate analysis using the Wilcoxon rank-sum statistic. NRS was collected at predetermined intervals at least 4 times in the first 8 hours after surgery. Nursing staff recorded hourly NRS in the cardiovascular intensive care units and every 4 hours in the cardiovascular intermediate care unit. Masked study coordinators queried participants on aggregated daily NRS and administered the BPI and 5-point satisfaction questionnaires once a day. Comparisons were made each day, and the AUC for the NRS over the cumulative 3-day period was also computed using the trapezoidal rule. If pain assessments were missed or patients were discharged before postoperative day 3, imputation of the nonmissing value nearest in time was used (last carried forward method). All supplementary analgesics, including both intravenous and oral opioids, were converted to standard morphine equivalent units (MEU) using a software tool developed in Oregon under a CDC cooperative agreement.^39^ Our service generally did not use nonsteroidal anti-inflammatory medications postoperatively because of the prevalence of kidney insufficiency in our patient population. We encouraged Dilaudid as our preferred opioid for breakthrough pain to simplify the analysis, but other opioids were not withheld if prescribed. Daily measures were compared using Wilcoxon rank-sum test and were further assessed for treatment-by-day interaction using nonparametric longitudinal mixed models with unstructured error terms. Main effects of day, treatment, and treatment-by-day interaction were modeled using fixed effects, with a random subject effect to account for within-subject clustering. For these models, *P* values are computed on ranked dependent variable data, and estimates are modeled using untransformed continuous values. The association between 72-hour pain score and opioid use was analyzed by fixed-effects generalized linear model with interaction. Lengths of stay for intensive care unit and total hospitalization were log-transformed for regression-based analysis but were analyzed by Wilcoxon rank-sum test for univariate comparisons, as were analgesics. If patients could not be assessed for pain because of prolonged intubation and sedation, they were excluded from the length-of-stay analysis. Complication frequencies were compared using contingency table tests, including the χ^2^ test where expected value assumptions were met and Fisher exact tests where expected cell frequencies were less than 5. *P* < .05 was considered significant and all tests were 2-sided. Data were analyzed using SAS statistical software version 9.4 (SAS Institute) from July to December 2018.

## Results

We randomized 338 individuals to reach 280 evaluable patients, with 140 assigned to each treatment, standard vs liposomal bupivacaine (Figure 1). Mean (SD) age was 60.2 (14.4) years, and 36% (101 of 280) were women. Mean (SD) incision length was 194.3 (96.4) mm. Pretreatment characteristics are presented in the Table.

**Table.** Characteristics and Results of Liposomal Bupivacaine Group vs Standard Bupivacaine Group^a^

Variable	Patients, No. (%)		RR (95% CI)^c^	*P* value^c^
	Liposomal bupivacaine (n = 140)^b^	Standard bupivacaine (n = 140)^b^		
Preoperative and baseline characteristics				
Age, mean (SD), y	60.3 (14.6)	60.1 (14.2)	NA	NA
Incision length, mean (SD), mm	201 (102.9)	187.7 (89.4)	NA	NA
Women	44 (31)	57 (41)	NA	NA
Prior				
Laparotomy	20 (14)	20 (14)	NA	NA
Thoracotomy	3 (2)	3 (2)	NA	NA
Sternotomy	18 (13)	10 (7)	NA	NA
Congestive heart failure	22 (16)	22 (16)	NA	NA
Known kidney disease	15 (11)	21 (15)	NA	NA
Coronary artery disease	77 (55)	80 (57)	NA	NA
Chronic obstructive pulmonary disease	22 (16)	18 (13)	NA	NA
Dyslipidemia	92 (66)	86 (61)	NA	NA
Hypertension	118 (84)	118 (84)	NA	NA
Diabetes	47 (34)	44 (31)	NA	NA
Body mass index, mean (SD)^d^	34.5 (45.0)	29.8 (9.4)	NA	NA
Baseline glomerular filtration rate, mL/min/1.73 m^2^	99.0 (50.2)	90.4 (40.4)	NA	NA
Chronic kidney disease stage			NA	NA
1	71 (51)	61 (43)		
2	39 (28)	50 (36)		
3	16 (11)	14 (10)		
3b	6 (4)	5 (4)		
4	3 (2)	3 (2)		
5	5 (4)	7 (5)		
Intraoperative and clinical outcomes				
Type of incision laparotomy	8 (6)	10 (7)	NA	NA
Minithoracotomy	19 (14)	18 (13)		
Sternotomy	98 (70)	99 (71)		
Thoracotomy	15 (11)	13 (9)		
Redo	15 (11)	9 (6)	1.39 (0.82-2.36)	NA
Extubated in OR	16 (11)	18 (13)	0.93 (0.67-1.32)	NA
Postoperation				
Kidney complications	16 (11)	17 (12)	0.97 (0.68-1.38)	.85
Cardiac complications	56 (40)	46 (33)	1.17 (0.91-1.51)	.21
Hypotension	70 (50)	66 (47)	1.06 (0.84-1.34)	.63
Infective complications	17 (12)	25 (18)	0.82 (0.61-1.08)	.18
Bleeding complications	34 (24)	33 (24)	1.02 (0.77-1.35)	.89
Vomiting	11 (8)	10 (7)	1.05 (0.66-1.68)	.82
Nausea	9 (6)	9 (6)	1.00 (0.62-1.61)	>.99
Gastrointestinal complications	19 (14)	20 (14)	0.97 (0.70-1.35)	.86
Wound complications	0	2 (1)	0.50 (0.44-0.56)	.50
ICU length of stay, d	3 (2-4)	3 (2-5)	NA	.91
Hospital length of stay, d	8 (6-13)	8 (6-12)	NA	.45
Postoperative pain scores				
NRS				
POD 1	5 (3-8)	5 (3.5-7)	NA	.70
POD 2	5 (3-6)	4 (2-6)	NA	.04
POD 3	3 (2-5)	3 (1-4.5)	NA	.08
Cumulative NRS (POD 1-3)	13.5 (9-17)	12 (8-16.5)	NA	.15
BPI: worst pain				
POD 1	9 (6-10)	8 (6-10)	NA	.54
POD 2	8 (5-9)	7 (5-9)	NA	.21
POD 3	6 (4-8)	5 (3-8)	NA	.11
BPI: least pain				
POD 1	3 (1-5)	3 (1-5)	NA	.38
POD 2	2 (0-4)	2 (0-4)	NA	.10
POD 3	1.5 (0-3)	0 (0-3)	NA	.07
BPI: average pain				
POD 1	5 (4-7)	5 (4-7)	NA	.97
POD 2	5 (3-6)	4 (2-6)	NA	.15
POD 3	4 (2-6)	3 (1-5)	NA	.049
BPI: pain right now				
POD 1	4 (2-7)	5 (2-7)	NA	.35
POD 2	4 (1-6)	3 (1-5)	NA	.12
POD 3	2 (0-5)	1 (0-4)	NA	.08
5-point satisfaction				
POD 1	4.5 (4-5)	5 (4-5)	NA	.93
POD 2	5 (4-5)	5 (4-5)	NA	.80
POD 3	5 (4-5)	5 (4-5)	NA	.21
Postoperative opioid consumption				
MEU, POD1	16.9 (8.3-33.4)	11.7 (5-25.7)	NA	.04
Dilaudid, mg, POD 1	0 (0-3.2)	0 (0-2.6)	NA	.63
Fentanyl, μg, POD 1	75 (25-175)	50 (25-137.5)	NA	.23
Morphine, mg, POD 1	0 (0-0.3)	0 (0-0.6)	NA	.86
Acetaminophen, mg, POD 1	1000 (0-3000)	1000 (0-2000)	NA	.26
MEU, POD 2	11.3 (3.4-20.9)	10.7 (2.9-22.5)	NA	.87
Dilaudid, mg, POD 2	0 (0-3.6)	0 (0-3.5)	NA	.53
Fentanyl, μg,POD 2	0 (0-0)	0 (0-0)	NA	.82
Morphine, mg, POD 2	10 (0-37.5)	10 (0-30)	NA	.51
Acetaminophen, mg, POD 2	1625 (650-3000)	1000 (325-2600)	NA	.01
MEU, POD 3	7.5 (1.9-13.5)	6.3 (1.9-11.7)	NA	.29
Dilaudid, mg, POD 3	0 (0-0.1)	0 (0-0)	NA	.07
Fentanyl, μg, POD 3	0 (0-0)	0 (0-0)	NA	.95
Morphine, mg, POD 3	15 (0-36.8)	20 (0-34.5)	NA	.70
Acetaminophen, mg, POD 3	1000 (0-2350)	1300 (325-1975)	NA	.56
MEU total	41.5 (21.3-73.8)	33 (17.8-62.5)	NA	.03

Abbreviations: BPI, brief pain inventory; ICU, intensive care unit; MEU, morphine equivalent units; NRS, numeric rating scale; OR, operating room; POD, postoperative day.

^a^ Continuous variables, including age, baseline glomerular filtration rate, body mass index, and incision length, are reported as mean (SD). Pain scores and opioid drug doses and morphine equivalent units are reported as median (interquartile range: 25th percentile to 75th percentile) with *P* values reporting nonparametric (Wilcoxon rank-sum) tests.

^b^ Categorical variables are reported as No. (%).

^c^ Measure of association refers to Wilcoxon *P* values for ordinal or nonnormally distributed variables, and risk ratio with 95% CIs is shown for categorical variables.

^d^ Body mass index is calculated as weight in kilograms divided by the square of height in meters.

All patients received their allocated treatment, and there were no follow-up losses in this hospital-based study. The most common reason for exclusion after randomization was prolonged intubation or reintubation after surgery; these patients were sedated such that pain scores could not be obtained. We paused enrollment at the end of 2013 because of staffing turnovers and began recruiting again in 2015 when staffing levels stabilized. The majority of patient recruitment was obtained from 2015 to 2017.

**Figure 2.**
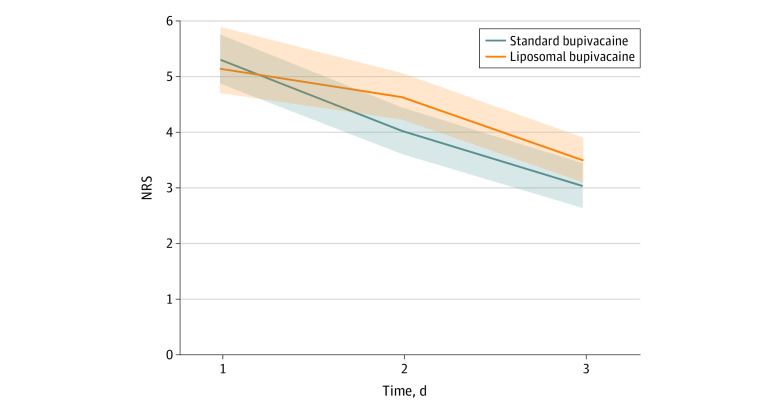
Numeric Rating Scale (NRS) Mixed Model NRS scores are shown by group over 3 days. No main effect of treatment (*P* = .23) was observed, but significant main effect of day (*P* < .001) and significant treatment-by-day interaction (*P* = .03) were present, indicating that rate of pain reduction was greater in the standard bupivacaine group over three postoperative days. Models shown are pain scores; *P* values are from mixed models of ranked data with unstructured error terms. Lines denotes regression function and shaded areas denote 95% CIs.

Six patients in the standard bupivacaine group and 3 in the liposomal bupivacaine group had a missing primary end point pain assessment (NRS) on 1 of the postoperative days, and those data points were imputed—a total of 9 data points imputed in 840 measurements (280 patients with 3 postoperative pain measurements each), for an imputation rate of approximately 1%. Two of those imputed were day 2 discharges, and the imputed day 3 score for the bupivacaine patient was 2 and for the liposomal bupivacaine patient was 0 (last observation carried forward). The median (interquartile range [IQR]) 3-day cumulative NRS was 12.0 (8.0-16.5) for bupivacaine and 13.5 (9.0-17.0) for liposomal bupivacaine (*P* = .15, Wilcoxon rank-sum). Daily values for the pain scales are shown in the Table. In general, according to unpaired daily comparisons, pain scale scores did not differ between groups over the 3-day time period. There was also no difference in satisfaction with pain control on the basis of the 5-point satisfaction questionnaire (Table). Nonparametric mixed models showed no significant main effect for standard vs liposomal (β = –0.46; SE = 0.29; *P* = .23) and a significant main effect for postoperative day (β = –0.87; SE = 0.11; *P* < .001), indicating that the treatments did not differ overall but that pain scores decreased significantly over the 3-day observation period. The term for treatment-by-day interaction was significant (*P* = .03), indicating that the decline in postoperative pain scores was more rapid in the standard bupivacaine group (Figure 2).

**Figure 3.**
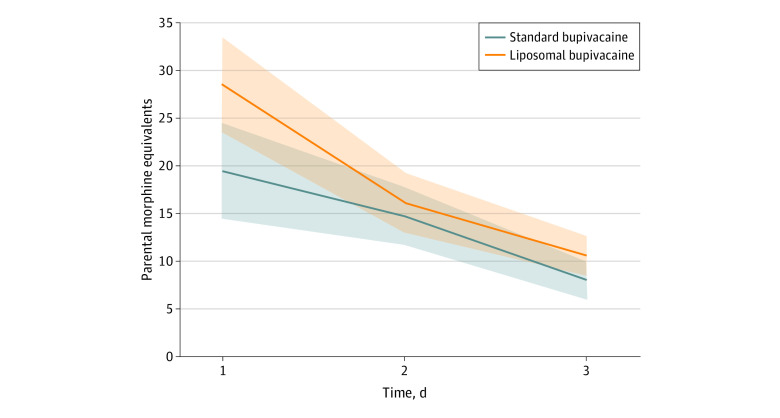
Opioid Use Mixed Model Opioid dose (parenteral morphine equivalents) is shown by group over 3 days postoperatively. No main effect of treatment (*P* = .12) or treatment-by-day interaction (*P* = .29) was observed, but a significant effect of day (*P* < .001) was. Hence, reduction in supplemental opioid use over 3 days is significant but does not depend on formulation of bupivacaine. Models shown are opioid doses; *P* values are from mixed models of ranked data with unstructured error terms. A pairwise contrast at day 1 is statistically significant (*P* = .04, Wilcoxon rank-sum). Lines denotes regression function and shaded areas denote 95% CIs.

Median (IQR) total opioid use was 33.0 (17.8-62.5) MEU in the standard bupivacaine group and 41.5 (21.3-73.8) MEU in the liposomal bupivacaine group (*P* = .03, Wilcoxon rank-sum) during 3 postoperative days. Daily values for supplemental opioid use are shown in the Table. In general, opioid use was not different between groups during the study period, although total opioid use and opioid use on postoperative day 1 was higher in the liposomal bupivacaine group (median [IQR], 16.9 [8.3-33.4] MEU vs 11.7 [5-25.7] MEU; *P* = .04, Wilcoxon rank-sum]. This effect faded by postoperative day 2 (11.3 [3.4-20.9] MEU vs 10.7 [2.9-22.5] MEU; *P* = .87, Wilcoxon rank-sum) and postoperative day 3 (7.5 [1.9-13.5] MEU vs 6.3 [1.9-11.7] MEU; *P* = .29, Wilcoxon rank-sum). In nonparametric mixed model analysis, the main effect of drug was not significant (standard vs liposomal, β = –2.62; SE = 1.45; *P* = .12), but the main effect of postoperative day was significant (β = –17.8; SE = 2.4; *P* < .001) and treatment-by-day interaction was not significant (*P* = .29). This indicates that treatment effects of supplemental opioid use did not differ between groups overall, that it did decline significantly over the 3 postoperative day observation period, and that the rate of decline between the treatment groups did not differ (Figure 3). One patient in each treatment group was discharged on postoperative day 2, but both were not taking any opioid pain medications at the time of discharge.

**Figure 4.**
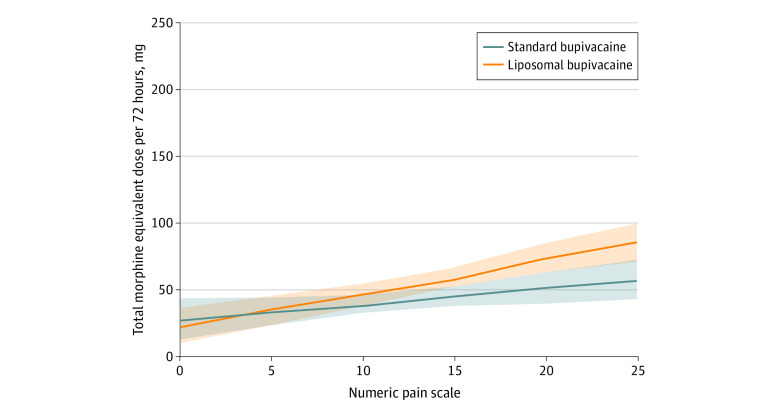
Effect of Cumulative Pain Rating on Cumulative Opioid Use—General Linear Model Opioid consumption is positively correlated with pain (*P* < .001), with pain accounting for approximately 10% of the variance in opioid use (multiple *R*^2^ = 0.109). Main effect of treatment is not significant. No modification of the effect by liposomal bupivacaine relative to standard bupivacaine is evident (*P* for interaction *P* = .08). Lines denotes regression function and shaded areas denote 95% CIs.

Pain score was associated with supplemental opioid use at all time points and accounted for slightly more than 10% of the variance overall. In general, linear model regression analysis, model terms for effect of pain score (SE) were significant (β = 2.56 [0.55] MEU/NRS unit; *P* < .001), but treatment group (β = 6.02 [11.12] MEU increase in standard vs liposomal; *P* = .59) and treatment-by-pain interaction (*P* = .08) were not statistically significant. This indicates that, although opioid use depended on perceived pain, the formulation of bupivacaine administered did not modify this association. In other words, liposomal bupivacaine did not significantly reduce opioid use for a given level of pain compared with standard bupivacaine (Figure 4).

Incision length did not differ between groups, and no incision length-by-treatment interaction was observed. Total NRS-reported pain was higher for thoracotomy incisions (thoracotomy and minithoracotomy combined) than the other incision types (14 [12-17] for thoracotomy vs 12 [8-17] for other incisions; *P* = .006, Wilcoxon rank-sum). Total opioid use was not different (38.8 [15.8-66.3] for thoracotomy vs 38.4 [20.0-68.1] for other incisions; *P* = .73, Wilcoxon rank-sum). Incision type was associated with pain with thoracotomy group reporting highest median (IQR) pain scores on postoperative days 1 (liposomal vs standard bupivacaine, 6 [4-8] vs 5 [3-7]; *P* = .049, Wilcoxon rank-sum) and 2 (liposomal vs standard bupivacaine, 5 [4-7] vs 4 [2-6]; *P* = .003, Wilcoxon rank-sum) but not day 3 (3 [2-6] vs 3 [1-5], *P* = .10, Wilcoxon rank-sum), irrespective of treatment group. No thoracotomy-by-treatment interaction was identified for pain (*P *for interaction = .06) or opioid use (*P* for interaction = .71). Because 70% of the incisions were sternotomies, we also performed a subgroup analysis within sternotomy and nonsternotomy groups. The findings were consistent with the overall findings of significant reduction in pain across the 3 postoperative days, but there were no differences in drug effect. In the nonsternotomy group (thoracotomy, minithoracotomy, and laparotomy), liposomal formulation was associated with less pain control than standard formulation (β = –1.14; SE = 0.57; *P* = .01), but no treatment-by-day interaction was identified.

No differences were observed in postoperative complications between the groups (Table). Neither intensive care unit length of stay nor hospital length of stay was significantly different between groups. There was 1 hospital death, which occurred in the standard formulation group.

## Discussion

Effective surgical pain control is an important treatment goal, reduces morbidity, and improves return to activity and to work.^26,27,28^ It is also a major patient-centered outcome and an important factor in patient satisfaction and quality of life. Increasingly, development and implementation of opioid-reducing pain management strategies is a substantial public health issue given the scope and scale of the opioid abuse crisis in the US. This is of particular concern for major truncal procedures. Several recent studies demonstrated that many patients are still using opioids many months after surgery.^29,30^ Studies have also shown that postsurgical exposure may increase addiction risk and that even family members of long-term opioid users may be at increased risk for long-term use after their own surgical procedures.^29,30,31,32,33^ Improved methods for controlling pain that can minimize opioid use in the postoperative setting are needed, and multimodal nonopioid pain control, including local analgesia, is an important element in a comprehensive pain management strategy.^27,34^

Epidural anesthesia can also play a role in certain truncal incisions, but it is not useful for sternotomies or superiorly placed thoracotomy incisions. Epidural anesthesia adversely affects neurological examination after open aortic surgery. Nevertheless, although our service does not routinely use epidural anesthesia, it can be a useful pain control adjunct in selected cases. Placement of thoracic epidural catheters typically does not reside with the surgical team. The advantage of surgeon-administered local anesthesia is that it is fast, easy, and available. We use local anesthesia as part of a successful multimodal regimen that includes nonopioid oral pain medications, gabapentin, locoregional nerve blocks, and dexmedetomidine infusion.^34^

Our goal was to determine whether liposomal bupivacaine would improve the intensity and duration of postoperative pain in major truncal surgery as it has been reported to do in other nontruncal orthopedic, cosmetic, and colorectal indications,^8,9,10,11,12,13,15,35^ and whether it could also reduce reliance on opioid medications. In this randomized clinical trial involving 280 patients with 4 different types of chest and abdominal incisions, which, to our knowledge, is the largest study of its kind yet to be reported, we were unable to identify any clinically important difference in pain, supplemental opioid use, morbidity, or length of stay between liposomal and standard formulations of bupivacaine. We did observe significant reductions in pain and opioid use in both groups over 3 postoperative days, and also found that the NRS scores were reduced at a more rapid rate in the bupivacaine HCl group (Figure 2). For major truncal surgery in the setting of a large academic medical center, the findings of this study do not support the hypothesized superiority of liposomal bupivacaine over standard bupivacaine HCl.

The literature on the efficacy of liposomal bupivacaine vs conventionally formulated bupivacaine is equivocal, with multiple publications concluding that liposomal bupivacaine is superior to standard bupivacaine, and others that it is no better. In one case, liposomal bupivacaine was no better even than placebo for the sternotomy indication with respect to supplemental opioid sparing.^15^ Reviews in the orthopedic surgery literature^36,37^ also concluded that liposomal bupivacaine performed no better than controls. A recent Cochrane review^38^ concluded that the quality of the literature was poor, and that the limited evidence available does not demonstrate superiority of liposomal bupivacaine over standard bupivacaine HCl. Our project was designed to be a clinical comparative effectiveness study, performed under typical surgical conditions in an academic medical center, and without industry support. The Cochrane review authors^38^ downgraded their assessment of evidence quality in assessment of liposomal bupivacaine relative to standard bupivacaine because of the small-sample treatment sets in most of the published studies and also the unclear risk of bias attributable to the financial ties of the research teams and the editorial process to the manufacturer. The Cochrane review authors^38^ also highlighted the disagreement between their review and two other previously published reviews^13,35^ and point out that this can be attributed to heterogeneity in research designs, surgical procedures and, again, financial relationships.

### Limitations

There were several limitations in the study. First, in the study design, we anticipated approximately equivalent numbers of incision types. In practice, we performed more sternotomies and fewer thoracotomies and laparotomies. This reflected a global trend toward increasing endovascular repair for thoracic and thoracoabdominal aortic aneurysms with fewer open aortic surgical procedures. Second, there is variability in technique, speed, and surgeon skill that may make our single-center results not fully replicable in other centers. The absolute pain scores are likely not generalizable because of different pain management regimens used by different groups. However, the comparison and effect size differences will still be useful. Third, although we attempted to standardize the administration of local anesthesia, there may be small differences between surgeons who performed more sternotomies compared with those who performed thoracotomies and laparotomies. Randomization and stratification by incision type should mitigate these limitations. Fourth, the postoperative pain assessments were done by coordinators masked to treatment assignment, but we could not blind the surgical team administering the local anesthesia. Liposomal bupivacaine has a white, milky appearance whereas standard bupivacaine is clear. Our institutional review board would not allow blinding of the operating room team because of concerns about confusing the study drugs and other drugs with the same appearance, such as propofol. Furthermore, local anesthesia was given at the conclusion of the operation. Some investigators preferred to administer local anesthesia prior to incision. We decided that this would complicate cases where the incision required lengthening for greater exposure. This should not affect the comparison because both groups received anesthesia in the same manner. This study did take longer than anticipated to conduct given the 2-year enrollment hiatus previously described. However, randomization was balanced by blocking every 4 to 6 participants, so any secular trends in pain management would have been absorbed equally into the treatment groups by design.

Fifth, some patients could not be evaluated after randomization because of unanticipated events such as prolonged intubation, so this is not strictly speaking an intent-to-treat analysis. The only randomized patients excluded from the analysis were those who did not have evaluable data, for example, because of prolonged intubation and inability to elicit pain scores. We would have included data for these patients if they existed. As a practical matter, a per protocol analysis is less conservative and, hence, reduces the likelihood of making a type 2 error, which in a negative study such as this would be the greater concern than the type 1 error intent-to-treat protocols are meant to guard against.

## Conclusions

The heterogeneity of the findings reported in the literature, and the low quality of the evidence either for or against the use of liposomal vs conventional formulations of bupivacaine, underscores the importance of independent comparative effectiveness research, performed with high methodological standards (randomized, masked designs with large enough samples to control small-sample bias) by independent teams of investigators. The results of this study do not support the use of the more expensive liposomal formulation over the standard formulation of bupivacaine for postoperative pain control in major truncal surgery.

## Notes

Supplement 1. **Trial Protocol**

Supplement 2. **Data Sharing Statement**
