# Supplementary material for: Effectiveness of Standard Local Anesthetic Bupivacaine and Liposomal Bupivacaine for Postoperative Pain Control in Patients Undergoing Truncal Incisions: A Randomized Clinical Trial
Source: JAMA Netw Open. 2021 Mar 16;4(3):e210753. doi: 10.1001/jamanetworkopen.2021.0753 (PMC7967071; doi:10.1001/jamanetworkopen.2021.0753)
Supplement: Supplement 1. — Trial Protocol [file jamanetwopen-e210753-s001.pdf]

Study Protocol

Table of Contents

I. LIST OF ABBREVIATIONS 3
II. PRINCIPAL INVESTIGATOR & RESEARCH TEAM 3
III. STUDY SITE 4
IV. CONTACT INFORMATION 4
V. RESEARCH SYNOPSIS 5
A. Study Title 5
B. Clinical Phase 5
C. Study Population 5
D. Study Design 5
E. Sample Size 5
F. Study Duration 5
G. Study drug and Intervention Description 6
H. Primary Objective 6
I. Secondary Objectives 6
VI. BACKGROUND AND SIGNIFICANCE 6
VII. PRIMARY OBJECTIVES 9
H. SECONDARY OBJECTIVES 11
IX. STUDY POPULATION 11
X. STUDY DRUGS 13
XI. STUDY SCHEDULE 13
XII. STUDY DESIGN/METHODOLOGY 14
Study Conduct 17
1. Screening 17
2. Quality of life assessment - Brief Pain Inventory 17
3. Enrollment 18
4. Randomization and Allocation Concealment 18
5. Preoperative assessment 19
6. Follow-up 19

UTHealth The University of Texas Health Science Center at Houston
IRB NUMBER: HSC-MS-13-0620
IRB APPROVAL DATE: 09/12/2016

7. Termination of enrollment 20
8. Termination of Study 20
XIII. ADVERSE EVENT REPORTING 20
XIV. STATISTICAL ANALYSIS PLAN 22
A. Primary Outcomes 22
A. Secondary Outcomes 23
B. Sample Size Determination 23
XV. ETHICS 25
B. Privacy and Confidentiality 26
C. Risk/Benefit 26
XVI. STUDY TIMELINE 27
XVII. DATA SAFETY MONITORING 28
XVIII. CONFLICT OF INTEREST 28
XIX. FUTURE PUBLICATION AND PRESENTATION 29
XX. References 29

UTHealth The University of Texas Health Science Center at Houston
IRB NUMBER: HSC-MS-13-0620
IRB APPROVAL DATE: 09/12/2016

I. LIST OF ABBREVIATIONS

AE	Adverse Effects
BPI	Brief Pain Inventory
CNS	Central Nervous System
FDA	Food and Drug Administration
LOS	Length of Stay
NPS	Numeric Pain Scale
NSAID	Nonsteroidal Anti-Inflammatory Drugs
MG	Milligrams
PCA	Patient Controlled Analgesia
PI	Principal Investigator

II. PRINCIPAL INVESTIGATOR & RESEARCH TEAM

Principal investigator: *Kristofer Charlton-Ouw, MD* (Vascular Surgeon)

Co-Investigators: Hazim Safi, MD (Cardiothoracic Surgeon)

Anthony Estrera, MD (Cardiothoracic Surgeon)

Tom C Nguyen, MD (Cardiothoracic Surgeon)

Philip A. Rascoe, MD (Cardiothoracic Surgeon)

Ali Azizzadeh, MD (Vascular Surgeon)

Kamal Khalil, MD (Cardiothoracic Surgeon)

Charles C. Miller, PhD (Biostatistician)

Research Team: Alexa Perlick, BS (Research Assistant)

UTHealth The University of Texas Health Science Center at Houston
IRB NUMBER: HSC-MS-13-0620
IRB APPROVAL DATE: 09/12/2016

Harleen Sandhu, MD MPH (Research Coordinator)

Samuel Leake, BS (Research Coordinator)

Harith Mushtaq, MD (Research Coordinator)

Jennifer Goodrick, RN (Research Nurse)

III. STUDY SITE

1. *Department of Cardiothoracic and Vascular Surgery*

   University of Texas Medical School at Houston

2. *Memorial Hermann Hospital – Texas Medical Center*

   Houston, Texas

IV. CONTACT INFORMATION

1. ***Kristofer Charlton-Ouw, MD***

   Principal Investigator
   6400 Fannin Street, Suite 2850, Houston, TX, 77030
   Phone (713) 486 – 5100 Fax (713) 512 – 7200
   Kristofer.CharltonOuw@uth.tmc.edu

2. ***Harleen Sandhu***

   Research *Coordinator*
   6400 Fannin Street, Suite 2850, Houston, TX, 77030
   Phone (713) 486 – 5131 Fax (713) 512 – 7200
   Harleen.k.sandhu@uth.tmc.edu

3. ***Alexa Perlick***

   ***Research* Assistant**
   6400 Fannin Street, Suite 2850, Houston, TX, 77030
   Phone (713) 486 – 5100 Fax (713) 512 – 7200
   Alexa.perlick@uth.tmc.edu

4. ***Samuel Leake***

   Research Coordinator

UTHealth The University of Texas Health Science Center at Houston
IRB NUMBER: HSC-MS-13-0620
IRB APPROVAL DATE: 09/12/2016

6400 Fannin Street, Suite 2850, Houston, TX, 77030
Phone (713) 486 – 5100 Fax (713) 512 – 7200
Samuel.S.Leake@uth.tmc.edu

V. RESEARCH SYNOPSIS

A. Study Title

Study Full Title

A prospective randomized-controlled study on effectiveness of extended-release liposomal bupivacaine in postoperative pain control.

Study Short Title

PAIN – Postoperative Analgesia INvestigation

B. Clinical Phase

Phase IV

C. Study Population

The study will include patients aged 18 and over who require sternotomy, thoracotomy, mini- thoracotomy (including Video Assisted Thoracoscopic Surgery (VATS) procedures), or laparotomy incisions.

D. Study Design

Prospective single-blinded, controlled randomized clinical trial with 2-armed parallel-group sequential design

E. Sample Size

330

F. Study Duration

4 years

UTHealth The University of Texas Health Science Center at Houston
IRB NUMBER: HSC-MS-13-0620
IRB APPROVAL DATE: 09/12/2016

G. Study drug and Intervention Description

Subjects will be randomized into two groups: group A will receive the study drug [bupivacaine liposomal injectable suspension (Exparel®)] and Patient Controlled Analgesia (PCA), and group B will receive regular bupivacaine hydrochloride (HCl) and PCA.

H. Primary Objective

To assess the efficacy of the intraoperative liposomal bupivacaine injection in postoperative pain control after truncal incisions..

I. Secondary Objectives

- *To compare opioid use between groups.*
- *To evaluate and compare patient participation in physical therapy.*
- *To evaluate and compare patient length of stay in hospital.*
- *To evaluate cost-effectiveness of adding extended-release local anesthetic to a postoperative pain control regimen.*

J. Clinical trials registration:

This trial has been registered at and the ClinicalTrials.gov Identifier is NCT02111746

VI. BACKGROUND AND SIGNIFICANCE

In the United States, around 48 million inpatient surgical procedures were performed in 2009[1]. Although pain is a predictable part of the postoperative experience (significant in up to 75% of patients [2, 3]), inadequate management is still common. Adequate postoperative pain management has shown to improve healing period, contribute to faster patient mobilization, and reduce the hospital length of stay and

UTHealth The University of Texas Health Science Center at Houston
IRB NUMBER: HSC-MS-13-0620
IRB APPROVAL DATE: 09/12/2016

healthcare cost.[4-6] Some studies show that the economic load of chronic pain that develops from an acute episode of pain in a 30 year-old patient may be as much as $1 million over a lifetime.[7] Lack of postoperative pain control may also result in deep vein thrombosis, pulmonary embolism, coronary ischemia, myocardial infarction, pneumonia, poor wound healing, insomnia and demoralization.[8,9] A number of drugs are available for postoperative pain control, such as opioids, nonsteroidal anti-inflammatory drugs (NSAIDS), local anesthetic agents infiltration (e.g., bupivacaine HCl), as well as combined regimens and protocols among them. Opioid use is the backbone of postoperative pain control but it does not come without risks. Severe opioid side-effects such as respiratory and central nervous system depression must be accounted for when assessing and managing pain. NSAIDs can affect integrity of gastric mucosa and overdose can cause renal impairment. Acetaminophen (Tylenol®) is generally safe but high doses can cause hepatic toxicity. One of the limitations of many pain control regimens, including most local anesthetic infiltrations, is that the effect usually lasts for less than 8 hours only.[10, 11]

Several drug delivery approaches and systems have been tested and are currently being used to prolong the duration and constancy of analgesic drug administration. The Patient Controlled Analgesia (PCA) method is one that is very commonly used in postoperative pain management protocols. This method was developed and introduced by Philip H. Sechzer in the late 1960s and described in 1971. It consists of allowing people to administer their own pain relief whenever they feel it is needed. On the surgical patient and while in hospital, the usual administration route is IV (intravenous), and the drugs usually used on a PCA system are from the opioid

UTHealth The University of Texas Health Science Center at Houston
IRB NUMBER: HSC-MS-13-0620
IRB APPROVAL DATE: 09/12/2016

family. The infusion is programmed by the prescriber in terms of demand dose, lockout interval, maximum drug delivery per hour, and rescue dose. For example, a morphine PCA could be set to deliver 1 mg of morphine sulfate on demand after a lockout interval of 10 minutes, a maximum cumulative dose of 8 mg per hour, and a rescue dose of an extra 2 mg to be given if there is inadequate pain control. If it is set and functioning as intended, the machine is unlikely to deliver an overdose of medication.

It is known that local anesthetics such as bupivacaine can achieve a good pain control, but is effective for only up to 8 hours. Recently, an injectable liposomal formulation of bupivacaine for extended-release (Exparel®) has been approved by the FDA. Exparel® has demonstrated to be effective in delivering bupivacaine for up to 72 hours.[12,13] Its liposome-based vehicle – DepoFoam®, ensures a slow yet continuous drug-release for a longer duration during the postoperative period as opposed to the shorter acting alternative of bupivacaine hydrochloride injection.[12,13]

Exparel® has shown a 30% pain reduction when compared to placebo.[12] The evidence comes from a few randomized controlled trials involving various types of surgeries, including hemorrhoidectomy, bunionectomy, mastectomy, orthopedic surgery, etc. At present, there is no study that analyzes its efficacy in truncal postoperative pain involving vascular, cardiac, laparotomy and/or thoracic surgical wounds. Such truncal wounds encompass sternotomy, thoracotomy, mini- thoracotomy (minimally invasive thoracic procedures, including, VATS), and laparotomy incisions. These fall under the regular practice of our group of surgeons. Postoperative pain management of these incisions can pose a difficult challenge. We propose a prospective, controlled and blinded randomized study to evaluate the effectiveness of

UTHealth The University of Texas Health Science Center at Houston
IRB NUMBER: HSC-MS-13-0620
IRB APPROVAL DATE: 09/12/2016

Exparel® for postoperative pain control. A prospective randomized study would allow an estimate on its efficacy and utilization as an option for the postoperative pain management for such operations.

VII. PRIMARY OBJECTIVES

The primary objective of the study is to compare the efficacy of standard local anesthetic bupivacaine and liposomal bupivicaine in postoperative pain control both in magnitude and duration in patients undergoing sternotomy, thoracotomy, mini-thoracotomy, and laparotomy incisions.

Specific **assessment tools** that will be used in our study:

1) Numeric Pain Scale (NPS): The NPS measures the intensity of pain. It consists of an 11-point scale from 0-10, where 0 means no pain and 10 means the most intense pain imaginable. Patients verbally select a value that correlates to the pain experienced by them. A written form can also be used. The NPS has good sensitivity and has a good capacity of producing data that can be statistically analyzed.[17,18,19,20]
2) Brief Pain Inventory (BPI): The Brief Pain Inventory is a medical questionnaire used to measure pain, developed by the Pain Research Group of the WHO Collaborating Centre for Symptom Evaluation in Cancer Care. Although the BPI is derived from the NPS it is more comprehensive and expanded. Besides assessing intensity of the pain, it provides a measure of effect of pain on the patient's life. The BPI is a powerful tool and has been tested for reliability and validity across cultures and languages. It is being adopted in many countries for

UTHealth The University of Texas Health Science Center at Houston
IRB NUMBER: HSC-MS-13-0620
IRB APPROVAL DATE: 09/12/2016

clinical pain assessment, epidemiological studies, and in studies evaluating the effectiveness of various pain treatments.[21,22]

3) Five-point satisfaction scale: This scale is used to assess the patient's pain management experience during their postoperative care. It consists of patients rating their overall postoperative pain management experience using a 5-point rating system. The scale ranges from 1 to 5, with 1 being "extremely dissatisfied", 2 "somewhat dissatisfied", 3 "Neutral/Neither satisfied nor dissatisfied", 4 "somewhat satisfied" and 5 "extremely satisfied" with their postoperative pain care. This rating has been used in other clinical trials where EXPAREL® analgesia was evaluated and it has proven to be a reproducible assessment tool.[12]

Primary outcome measures

Numeric Pain Scale (NPS) measurements will be assessed through 72 hours after the intraoperative injection. Each subject, using the numeric scale from 0 to 10 (11 numbers, 0 being no pain, and 10 the highest pain) will self-assess pain level daily at 9 a.m. (± 3 hours) beginning on post-operative day (POD) 1 through POD 3. The collected NPS measurements will be statistically transformed into rank scores which will be used to analyze data between groups.

Brief Pain Inventory (BPI) – Each subject will be asked to complete the BPI before the procedure and at 9 a.m. (± 3 hours) thereafter beginning on POD 1 through POD 3..

The 5-point satisfaction – Each subject, using a 5 number (1 to 5) scale will be assessed on post-surgical analgesia satisfaction (from 1 being "very dissatisfied" to 5

UTHealth The University of Texas Health Science Center at Houston
IRB NUMBER: HSC-MS-13-0620
IRB APPROVAL DATE: 09/12/2016

being “very satisfied”) at the same time as NPS and BPI are recorded (daily at 9 a.m. (± 3 hours) beginning on POD 1 through POD 3.)

H. SECONDARY OBJECTIVES

- To compare opioid use between groups.
- To evaluate and compare patient participation in physical therapy
- To evaluate and compare patient length of stay in hospital.
- To evaluate cost-effectiveness of adding extended-release local anesthesic to postoperative pain control regimen.

Secondary outcome measures

- The total amount in milligrams of opioid medication consumed through 12, 24, 36, 48, 60 and 72 hours after surgery.
- Patient participation during physical therapy rehabilitation duration to reach an optimal functional level
- Length of hospital stay
- Hospital cost for patient care during hospitalization will be estimated from hospital charges and financial records.

IX. STUDY POPULATION

The target population of our study will be comprised of patients that are 18 years-old and above that present to our group at The University of Texas Medical School at

UTHealth The University of Texas Health Science Center at Houston
IRB NUMBER: HSC-MS-13-0620
IRB APPROVAL DATE: 09/12/2016

Houston and will require at least one of the following: median sternotomy thoracotomy, mini-thoracotomy (minimally invasive thoracic procedures, including, VATS), or midline laparotomy. Our plan is to enroll 330 patients over a period of 4 years.

Inclusion/Exclusion Criteria

Patients will be *eligible for the study if*:

- 18 years-old or older, and
- Sternotomy, thoracotomy, mini-thoracotomy, or laparotomy incision is planned
- There is reasonable expectation that the patient will be extubated within 24 hours after surgery

Patients will be *excluded from the study if*:

- The patient has a known allergy to morphine or any opioid
- The patient has a known chronic pain disorder or takes daily opioid medication > 1 month prior to surgery
- There is anticipated difficulty communicating pain status due to language or other barriers at the investigator discretion.
- High postoperative morbidity index based on preoperative assessment, such as, low likelihood of extubation within 24 hours, extensive thoracoabdominal aortic aneurysm (Extent 2 TAAA), preoperative renal insufficiency/failure, etc.

Protocol Deviation:

- If during the first 72 hours after surgery, patients receive non opioid analgesics.

UTHealth
The University of Texas
Health Science Center at Houston
IRB NUMBER: HSC-MS-13-0620
IRB APPROVAL DATE: 09/12/2016

- If the patient was not given the scheduled drug intraoperatively due to unforeseen intraoperative situations, such as, prolonged operative time, delayed formal wound closure, etc.

Withdrawal criteria:

- Voluntary: patients who had consented and enrolled in the trial will maintain their right to withdraw at any point during the study as explained in the informed consent.

- In case of unexpected/unpredicted events, the Principal Investigator and research staff will analyze if patient meets criteria for withdrawal.

X. STUDY DRUGS

Exparel® is an FDA-approved bupivacaine liposome injectable suspension produced by Pacira Pharmaceuticals. Bupivacaine HCl is an FDA-approved injectable suspension. The standard non-liposomal bupivacaine is from Hospira pharmaceuticals.

XI. STUDY SCHEDULE

Expected start date after protocol amendment 1: October 1st 2014

Expected end date for enrollment: October 30th 2016

Length of enrollment for each patient: 3 days

Length of follow-up for each patient: 3 days after surgical procedure. A passive follow-up using review of electronic medical records for safety at 30 days.

UTHealth The University of Texas Health Science Center at Houston
IRB NUMBER: HSC-MS-13-0620
IRB APPROVAL DATE: 09/12/2016

XII. STUDY DESIGN/METHODOLOGY

This will be a single institution, prospective, blinded, randomized, controlled effectiveness study with 2-armed parallel-group sequential design to assess the efficacy of liposomal bupivicaine in postoperative pain control when compared to regular bupivacaine HCl. Exparel® liposomal bupivicaine is an FDA-approved suspension consisting of a liposomal form of the local anesthetic bupivacaine; it is to be injected intraoperatively in the surgical wound just prior to closing the incision.

Screened patients meeting the eligibility criteria will be randomized into one of the two study groups, i.e., liposomal bupivacaine and regular non-liposomal bupivacaine HCl injection into the surgical wound. Patients in both groups will receive the standard opioid PCA protocol during their postoperative period. Patients will receive a single dose of intravenous acetaminophen perioperatively as per the standard practice by our anesthesiologists.

During the screening and consenting process prior to surgery in the preoperative holding area, the inpatient wards, or in the outpatient clinic, patients will be educated on PCA use and the NPS and BPI assessments. Their preoperative baseline pain level, using BPI will be recorded at this time.

Patients from both groups will receive the same volume, 80ml, which will be given in four 20ml syringes using 22 gauge needles. The study drug group, will receive 266mg of liposomal bupivicaine (equivalent of one 1.3% 20ml vial of EXPAREL®) diluted in 60ml of preservative-free normal (0.9%) sterile saline for a total volume of 80ml. The non-liposomal bupivacaine HCl group, will receive 125mg of bupivacaine

UTHealth The University of Texas Health Science Center at Houston
IRB NUMBER: HSC-MS-13-0620
IRB APPROVAL DATE: 09/12/2016

hydrochloride (equivalent of one 0.25% 50ml or five 0.25% 10ml vials) diluted in 30ml of preservative-free normal (0.9%) sterile saline for a total volume of 80ml. Study drug or Bupivacaine HCl infiltration will be performed at the end of the procedure, just prior to wound closure. The surgical wound length will be measured.

Patients will receive an injection along their incision. Patients that require thoracotomies, mini-thoracotomies (minimally invasive thoracic procedures, including, VATS), and sternotomies will undergo serial intercostal nerve blocks. Intercostal nerve block will be done injecting 5cc (of the total 80cc) on each side of the incision on each intercostal space. The nerve blocks should be done on all intercostal spaces that compromise the incision. For patients undergoing VATS, the main thoracic surgical incision will be covered with a nerve block as previously explained for thoracotomies/mini-thoracotomies or sternotomies, while all additional incisions (typically 2-4 cm) will need drug infiltration if outside the region covered by nerve block. In addition, all patients (including all surgical incisions) will be injected in the fascia, subcutaneous tissues, periosteum (if applicable) and parietal pleura (if applicable). The total volume injected will be 80cc regardless of incision type.

All patients will be blinded to the contents of the intraoperative injection. The surgical team will be blinded up until the time of injection following which the knowledge of drug is inevitable since Exparel® has a milky appearance as opposed to normal HCl bupivacaine, which is colorless.

Patients will have access to the standard PCA (Patient Controlled Analgesia) offered at the Memorial Hermann Hospital – Texas Medical Center. The PCA drug will

UTHealth The University of Texas Health Science Center at Houston
IRB NUMBER: HSC-MS-13-0620
IRB APPROVAL DATE: 09/12/2016

be Dilaudid (hydromorphone). Initial dosing will be per the standard hospital protocol of 0.2 mg demand dose, 10 minute lockout, 2 mg per hour max, 0.4 mg rescue dose. Adjustments to the PCA dosing will be made based on clinical needs. Patients will receive PCA until the third postoperative day, and at that point, PCA will be switched to an oral analgesic, at the health-care team discretion. Modifications to the PCA regimen can be made by the health care team based on clinical need and will not be deemed a protocol deviation.

Per the standards of care at our institution, pain assessment is performed periodically by the nursing staff in the post-operative period using the Numeric Pain Scale. Formal NPS will be recorded by the research personnel daily at 9 a.m. (± 3 hours) beginning on post-operative day (POD) 1 through POD 3. NPS will also be recorded right before the first use of opioid rescue medication postoperatively (if applicable and if NPS had not been assessed within the last hour).

Patients in the study should also complete the Brief Pain Inventory (BPI) before the procedure (at baseline during the screening process) and daily postoperatively at 9 a.m. (± 3 hours) beginning on POD 1 through POD 3. Additionally, patients will be assessed on their satisfaction with the postsurgical analgesia. This will be done using the 5-point satisfaction scale with 1 being “very dissatisfied” to 5 being “very satisfied” daily postoperatively at 9 a.m. (± 3 hours) beginning on POD 1 through POD 3. After the 3 days postoperative monitoring and BPI reporting, the patient terminates his/her enrollment. This enrollment termination does not mean severance of the physician-patient relationship. In addition, a passive follow-up for safety will be performed that will involve review of electronic medical records for complications or any readmissions.

UTHealth The University of Texas Health Science Center at Houston
IRB NUMBER: HSC-MS-13-0620
IRB APPROVAL DATE: 09/12/2016

Patients will be consented and their medical records pertinent to the study will be collected on a case report form and this information will be kept on a secured HIPPA-compliant web-accessed database. No protected health information (PHI) will be maintained on the web-accessible database.

Study Conduct

1. Screening

The research group will assess patients as possible candidates for enrollment based on the inclusion criteria and the planned surgical exposure: median sternotomy, thoracotomy, midline laparotomy, and/or mini- thoracotomy (including VATS) incision. Once the eligibility is met, an informed written consent will be collected. A member of the research team - the PI, Co-PI, research coordinator, or nurse will obtain the consent from the surgical candidates who met the preliminary inclusion criteria. The consent process will take place either during the preoperative clinic visit, upon inpatient admission, or in the preoperative holding area. Patients will be consented before their surgical procedures and will be given the appropriate time to think and discuss with family about being part of the study. There will also be given enough time for the patients to ask questions about the protocol and research study. Should the patient decide to participate, patients will be educated on PCA use and the NPS and BPI assessments. Their preoperative baseline pain level, using BPI will be recorded at this time. A copy of the signed informed consent shall be provided to the patient, a copy will be placed in the patient’s hospital chart, and a copy will be kept by the research team. Medical/surgical history, vital signs measurements, drug/alcohol screening, and

UTHealth The University of Texas Health Science Center at Houston
IRB NUMBER: HSC-MS-13-0620
IRB APPROVAL DATE: 09/12/2016

standard preoperative testing will also be performed as per the usual surgical practice.

2. Quality of life assessment - Brief Pain Inventory

The impact of pain on patient's quality of life will be assessed through a brief pain inventory (BPI) that should be done before surgery and daily postoperatively at 9 a.m. (± 3 hours) beginning on POD 1 through POD 3. The BPI will be presented as an easy to comprehend questionnaire for the patients to quantify and communicate their pain experience and how it is affecting their life. It is designed such that patients with 6th-grade education should be able to complete the questionnaire on their own. Question and answers will be provided in a check-box format. The BPI that will be used on this study will be the short version. Patients with difficulty reading or who are physically unable to complete the questionnaire will be assisted by the research team based on their verbal responses to the BPI questions.

3. Enrollment

After screening for eligibility, if all inclusion/exclusion criteria are met and the patient has consented; the patient will be enrolled in the trial. At this point, the study coordinator/research office will be notified so that the patient can be randomly allocated to one of the study groups.

4. Randomization and Allocation Concealment

Randomization and allocation concealment will be performed via a web-based/computer generated block randomization sequence/list secured in a password-protected secure computer in the trial office accessible only to the research staff

UTHealth The University of Texas Health Science Center at Houston
IRB NUMBER: HSC-MS-13-0620
IRB APPROVAL DATE: 09/12/2016

independent of the trial administration process, who are not involved in the recruitment, data collection, analysis, assessment and/or follow-up. This office/research staff will be contacted by the blinded research assistant/investigator to provide certain specific details of the patient to be randomized that will be entered in the secure (password protected) computed-based entry system. The patient would then be allotted a trial or study number and allocated to a study arm group as per the randomized sequence list. Masking will include the operating surgeon who shall be blinded to the allocation process until the time of injection following which the knowledge regarding the drug to the administering surgeon is inevitable as the color of liposomal/extended release bupivacaine (study drug) is milky-white as opposed to the colorless bupivacaine hydrochloride (standard) formulations.

The randomization will be designed to balance the groups at 94 and 188 total subjects to maximize statistical efficiency. We will use block randomization to achieve a patient allocation ratio of 1:1, using varying blocks of 4 as per a web-based/computer generated list. Patients will be randomly allocated based on this permuted sequence to either of the two study groups.

5. Preoperative assessment

Pain assessment will be also done using the BPI form.

6. Follow-up

After the surgical procedure is done, the follow-up will be:

i. 9 am (+ or - 3 hours) of the first post-op day the NPS, BPI, and 5-point

UTHealth The University of Texas Health Science Center at Houston
IRB NUMBER: HSC-MS-13-0620
IRB APPROVAL DATE: 09/12/2016

scale will be assessed

ii. *9 am (+ or - 3 hours) of the second post-op day the NPS, BPI, and 5-point scale will be assessed*

iii. *9 am (+ or - 3 hours) of the third post-op day the NPS, BPI, and 5-point scale will be assessed*

7. Termination of enrollment

Patient will be considered to have his/her enrollment terminated:

i. *After the end of 3 days postoperatively*

ii. *If any medical/surgical/environmental condition develops that would affect or impair primary or secondary outcomes measurements results*

iii. *If the study is terminated*

iv. *If the patient dies*

8. Termination of Study

i. *End of study period*

ii. *If a clinically/statistically significant difference is achieved early during an interim analysis.*

XIII. ADVERSE EVENT REPORTING

UTHealth The University of Texas Health Science Center at Houston
IRB NUMBER: HSC-MS-13-0620
IRB APPROVAL DATE: 09/12/2016

Patients enrolled in the trial will be informed to report any unwanted effect at any time during the study. Adverse effects of bupivacaine will be made plain to the patients during the screening/enrollment as well as in the informed consent process. Most adverse side effects are related to incorrect drug administration technique, resulting in systemic exposure to higher blood plasma levels. This incorrect drug administration is extremely rare amongst trained and board certified vascular surgeons. All the surgeons involved in our study have been extensively trained on the administration technique required for local anesthetic injections. Bupivacaine can be cardiotoxic and amongst its side effects it can cause hypotension, bradycardia, arrhythmias and/or cardiac arrest. Other adverse effects include symptoms of central nervous system excitation (nervousness, tingling around the mouth, tinnitus, tremor, dizziness, blurred vision, seizures) or depression (such as drowsiness, loss of consciousness, respiratory depression and apnea).

<u>Safety monitoring will include assessment of:</u>

i. Adverse Effects (AE)
ii. Vital Signs
iii. Wound healing status
iv. Scarring
v. Cardiac monitoring

All aforementioned parameters will be closely monitored in a continuous manner, as part of our standard postoperative care. Any abnormalities found will be promptly assessed and described in the patient's medical records. Once the patient is discharged it is very unlikely that any of the study drugs will cause any abnormalities. Several times

UTHealth The University of Texas Health Science Center at Houston
IRB NUMBER: HSC-MS-13-0620
IRB APPROVAL DATE: 09/12/2016

the half-life of both the study group and HCl bupivacaine will have passed before the patient is discharged. Despite this fact, patients will be asked to contact the hospital in case they feel anything unusual. Definitions and examples of the standard or known adverse events associated with Exparel® and HCl bupivacaine like restlessness, anxiety, dizziness, tinnitus, blurred vision, tremors, convulsions, hypotension, bradycardia, ventricular arrhythmias, etc., will be presented to the patient for reference. Patients will be also assessed on the above items on their postoperative clinic visits. In addition, emergency contact information of the office in order to speak to someone regarding documentation of these adverse events will be provided. If need be, an immediate unscheduled visit shall be arranged if the patient so desires.

The PI will review AE as they occur, changes to the study design or drug use will be determined on an ongoing basis. AE will also be reported to the DSMB.

XIV. STATISTICAL ANALYSIS PLAN

A. Primary Outcomes

NPS measurements will be collected for each subject over the initial 72 hours postoperatively and the primary endpoint will be measured by a statistical comparison using a Wilcoxon rank-sum test. NPS measurements are derived from a likert scale that does not follow a normal distribution. Consequently, NPS measurements will be transformed to rank scores and analyzed by Wilcoxon rank-sum test between groups at each time point. Summary measures will be by two-way non-parametric (Friedman) ANOVA to assess time-by-group interaction over the first

UTHealth The University of Texas Health Science Center at Houston
IRB NUMBER: HSC-MS-13-0620
IRB APPROVAL DATE: 09/12/2016

three postoperative days. Randomization is expected to balance the groups with respect to preoperative risk factors.

A. **Secondary Outcomes**

Cumulative dose of opioid medication will be assessed as the sum of morphine doses dispensed by PCA pump during the first 72 hours. This will be compared by unpaired t test.

Length of hospital stay will be transformed to log scale to normalize the distribution if necessary and will be compared between groups by an appropriate unpaired statistic.

Attainment of physical therapy goal that justifies discharge from inpatient PT within 72 hours will be assessed by an appropriate contingency table test.

B. **Sample Size Determination**

Our surgical service manages many different types of patients but utilizes four major incisions (sternotomy, thoracotomy, laparotomy and mini- thoracotomy (including VATS)) to achieve surgical exposure. While some variation in discomfort associated with each procedure is likely to exist, we propose to power the study for aggregate effect on the entire group rather than to quadruple (or more, depending on the analytical framework) the sample size to be able to look definitively at each individual incision type. A complex incision-by-incision study would take years to accomplish and involve more than a thousand patients. A reasonable answer that could improve patient care may be available sooner, with a far less detailed and

UTHealth The University of Texas Health Science Center at Houston
IRB NUMBER: HSC-MS-13-0620
IRB APPROVAL DATE: 09/12/2016

resource-intensive methodology. The current dogma in the surgical community is that thoracotomies are the most painful and sternotomies the least painful of these incision types, but quantitative data sufficient to plan sample size on this basis are lacking. Currently, the best available data on postoperative pain reduction with extended-release-Bupivacaine are from a randomized study after hemorrhoidectomy that reports cumulative pain scores as mean areas under the NRS curve of 141.8 and 202.5 with a standard error of 10.7 in the Exparel® and HCl bupivacaine group, respectively.[12] The Cohen's delta effect size in this paper was 0.54, which is empirically determined to be a "medium sized" effect.[15]

Because we expect that Bupivacaine-related effect sizes might be larger in thoracotomies (which are considered more painful) than sternotomies, we have reduced our aggregate effect size expectation to a Cohen delta of 0.35, which is at the low end of the "medium" range but still clinically meaningful as it corresponds to a treatment group non-overlap of about 20% (based on the statistical properties of the distributions involved). As a hedge against the possibility that our assumptions are too conservative, we propose a group-sequential design [14] with three looks. This design confers the added benefit of ability to stop early for efficacy or harm (the alpha spending function is two-tailed) if an unexpectedly large signal is detected prior to full enrollment.

A total sample size of 330 subjects (165 Exparel®, 165 HCl bupivacaine) at cumulative alpha = 0.05 yields a beta of 0.17. The first interim look at 94 subjects requires a nominal alpha of 0.0002 to reject the null hypothesis. The second look at 188 patients puts the nominal critical value of alpha at 0.012. The final alpha has to

UTHealth The University of Texas Health Science Center at Houston
IRB NUMBER: HSC-MS-13-0620
IRB APPROVAL DATE: 09/12/2016

reach 0.0462 to aggregate total alpha to 0.05 if the study goes all the way to the full 330 cohort size. We think a conservative aggregate effect size estimate (Cohen 0.35) with two interim looks provides the best balance of design efficiency and coverage of the unknown probability space of the multiple incision effect sizes.

XV. ETHICS

A. Informed Consent Process

All eligible patients who meet the inclusion criteria will be consented, if willing, after an in-depth description of the study, the study drug and its adverse effects, the risks and benefits, follow-up routine, enrollment and termination terms. The importance of follow-up will be emphasized; however, the patient will retain the right to voluntarily withdraw from the study at any point. It will also be stressed that patient confidentiality will be kept at all times during study through de-identification of data.

Information will be stated clearly in a written consent form that will be designed at a 6th grade level of understanding. Information will also be presented verbally. A member of the research team, either the PI, Co-PI, or co-investigators will obtain the consent of the patient or other the legally authorized representative of the patient. The consent process will take place either during the preoperative clinic visit, upon inpatient admission, or in the preoperative holding area. The member of the team who is obtaining the written consent will describe the research project in its entirety and answer the questions of the patient and/or family members should they ask any. Sufficient time will be provided to the patient party to review the study before making an informed decision to participate or decline. We will make sure the patient have time to deliberate

UTHealth The University of Texas Health Science Center at Houston
IRB NUMBER: HSC-MS-13-0620
IRB APPROVAL DATE: 09/12/2016

on the risk/benefits of joining the study. If they require, patients will be given at least one hour for deliberation and discussion with the research staff, friends and/or family members about enrolling or not the study Should they decide to participate, three copies of the consent form would be attested by the patient or legally authorized individual for the patient's medical record, the study record, and one copy will be provided to the patient.

B. Privacy and Confidentiality

The study will be conducted in compliance with all HIPAA guidelines to protect patient confidentiality. All sensitive information or patient identifiers will be stored in form of a patient linkage file that will link the patient study/trial number to their clinical records and secured on the Zone 100 drive on specific networked computers of our department. We have a password protected electronic database that, along with the case report forms, will be de-identified and contain only study relevant data points and the patient's trial number. Access to any data pertaining to the study will be restricted to approved research team members, the FDA, institutional review boards of the University of Texas and Memorial Hermann Hospital.

C. Risk/Benefit

Risks to participants

There is a small increased risk to the patients by participating in the study in addition to a possible chance of breach in patient confidentiality as the study involves evaluation of an FDA-approved drug. As detailed in the informed consent, the subjects are at risk of

UTHealth The University of Texas Health Science Center at Houston
IRB NUMBER: HSC-MS-13-0620
IRB APPROVAL DATE: 09/12/2016

developing adverse effects from Exparel®, described on the drug packet insert and from HCl Bupivacaine, also described on the drug packet insert. Patients will be offered standard optimal medical therapy options, even if that will culminate in withdrawal from the study. In addition, there is risk of an unintentional disclosure of personal health information.

Benefits to participants

The patients stand to receive no direct benefit from the study. However, in the event our results endorse the published reports of reduction in postoperative pain by Exparel®, the group of patients receiving it might be indirectly benefitted through participation.

XVI. STUDY TIMELINE

Considering our group's monthly average for the given procedures and the enrollment rates, drop-outs and withdrawal, and interim analyses we estimate recruitment of our sample size will take 3 years. Once the follow-up of all enrolled participants is finished, data will be analyzed and published. The following is a chronological estimate of the stages:

Stage 1: Patient screening and enrollment	0 - 3 years
Stage 2: Follow-up	(0 – 3 years) No long-term follow-up relevant
Stage 3: Data collection and analysis	1 year

Stage 4: Presentation and publication period (at the end of data analysis)

UTHealth The University of Texas Health Science Center at Houston
IRB NUMBER: HSC-MS-13-0620
IRB APPROVAL DATE: 09/12/2016

XVII. DATA SAFETY MONITORING

In view of the uncertainty attached to the treatment effects in this heterogeneous population, owing to limited currently available data, an independent Data Safety Monitoring Committee, will oversee the progress and:

i. Monitor avoidable risks associated with patient data and confidentiality.

ii. Monitor achievement of trial recruitment targets, appropriateness of trial size,

iii. Evaluate interim analysis and advise remedial actions in case of superiority/futility situations

Two interim analyses will be conducted after the enrollment of the 94th and 188th patient, respectively using a group-sequential design. During these interim assessments the DSMC will decide on need for continuation based on the evidence that a reliable conclusion can be drawn based on available data (superiority) or unlikely to observe a statistical difference (futility). As for instance, if one between-group difference is demonstrably inferior to the other using a three-stage group-sequential overall nominal value of 0.05 [14] (alpha spending of 0.0002, 0.012 and .0462 at 94, 188 and 330 participants accrued, respectively) the study will be stopped early and the results will be reported to the IRB and published.

XVIII. CONFLICT OF INTEREST

There is no relationship between the PI or any other research staff member and Pacira

UTHealth The University of Texas Health Science Center at Houston
IRB NUMBER: HSC-MS-13-0620
IRB APPROVAL DATE: 09/12/2016

pharmaceuticals.

XIX. FUTURE PUBLICATION AND PRESENTATION

The results of this study will be analyzed and published after the approval of the principal investigator and biostatistician in a peer-reviewed scientific journal and/or presented at an international/national scientific conference or meeting regardless of outcome. The publication will acknowledge members of the study research group for their contributions and will maintain patient data protection.

XX. REFERENCES

1. Stats. National Center for Health Statistics Web site. Available at: http://www.cdc.gov/nchs/fastats. Accessed April 13, 2012
2. American Society of Anesthesiologists Task Force on Acute Pain Management.
3. Schug S, Large R. Economic considerations in pain management. Pharmacoeconomics 1993;3:260-7
4. Practice guidelines for acute pain management in the perioperative setting: an updated report by the American Society of Anesthesiologists Task Force on Acute
5. Pain Management. Anesthesiology. 2004; 100:1573-1581.
6. Crews JC. Epidural opioid analgesia. Crit Care Clin 1990;6:315-342
7. Cousins MJ, Power I , Smith G. Pain: a persistent problem. Reg Anesth Med 2000;25:6-21
8. Carr DB, Goudas LC. Acute pain. Lancet 1999;353:2051-8
9. Brevik H. Posoperative pain management: why is it difficult to show that it improves outcome? Eur J Anaesthesiol 1998;15:748-51
10. Vinson-Bonnet B, Coltar JC, Fingerhut A, Bonnet F. Local infiltration with ropivacaine improves immediate postoperative pain control after hemorrhoidal surgery. Dis Colon Rectum. 2002;45:104-108

UTHealth The University of Texas Health Science Center at Houston
IRB NUMBER: HSC-MS-13-0620
IRB APPROVAL DATE: 09/12/2016

11. Lohsiriwat D, Lohsiriwat V. Outpatient hemmorrhoidectomy under perianal anesthetics infiltration. J Med Assoc Thai. 2005;88:1821-1824
12. Gorfine SR, Onel E, Patou G, Krivokapic ZV. Bupivacaine Extended-Release Liposome Injection for Prolonged Postsurgical Analgesia in Patients Undergoing Hemorrhoidectomy: A multicenter, randomized, double-blind, placebo-controlled trial. Diseases of the Colon & Rectum. 2011;54(12):1552-1559
13. Bramlett K, Onel E, Viscusi ER, Jones K. A randomized, double-blind, dose-ranging study comparing wound infiltration of DepoFoam bupivacaine, an extended-release liposomal bupivacaine, to bupivacaine HCl for postsurgical analgesia in total knee arthrosplasty. The Knee 2012. IN PRESS
14. O'Brien, P. C., & Fleming, T. R. A multiple testing procedure for clinical trials. Biometrics. 1979; 35(3), 549-556. JSTOR.
15. Cohen, J. (1988). Statistical power analysis for the behavioral sciences (2nd ed.) Hillsdale, NJ: Lawrence Earlbaum Associates.
16. Patient controlled analgesia for adults. Thomson Healthcare, Inc. 2010.
17. Jensen MP, Karoly P, O'Riordan EF, Bland F, Burns RS. The subjective experience of acute pain. An assessment of the utility of 10 indices. Clin J Pain. 1989; **5** (2): 153–9.
18. Hartrick CT, Kovan JP, Shapiro S (December 2003). The numeric rating scale for clinical pain measurement: a ratio measure? Pain Pract. 2003; **3**(4): 310–6.
19. Williamson, A. and Hoggart, B. Pain: a review of three commonly used pain rating scales. J Clin Nurs 2005; 14: 798-804
20. Stratford, P. and Spadoni, G. The reliability, consistency, and clinical application of a numeric pain rating scale. Physiotherapy Canada. 2001; 53(2): 88-91.
21. Cleeland CS, Ryan KM (March 1994). Pain assessment: global use of the Brief Pain Inventory". Ann. Acad. Med. Singap. 1994; **23** (2): 129–38.
22. Caraceni A, Cherny N, Fainsinger R, *et al*. Pain measurement tools and methods in clinical research in palliative care: recommendations of an Expert Working Group of the European Association of Palliative Care. J Pain Symptom Manage. 2002; **23** (3): 239–55.

UTHealth The University of Texas Health Science Center at Houston
IRB NUMBER: HSC-MS-13-0620
IRB APPROVAL DATE: 09/12/2016
