# Supplementary material for: Effectiveness of Standard Local Anesthetic Bupivacaine and Liposomal Bupivacaine for Postoperative Pain Control in Patients Undergoing Truncal Incisions: A Randomized Clinical Trial
Source: JAMA Netw Open. 2021 Mar 16;4(3):e210753. doi: 10.1001/jamanetworkopen.2021.0753 (PMC7967071; doi:10.1001/jamanetworkopen.2021.0753)
Supplement: Supplement 2. — Data Sharing Statement [file jamanetwopen-e210753-s002.pdf]

## Data Sharing Statement

**Sandhu HK, Miller III CC, Tanaka A, Estrera AL, Charlton-Ouw KM. Effectiveness of standard local anesthetic bupivacaine and liposomal bupivacaine in postoperative pain control in patients undergoing truncal incisions. *JAMA Netw Open*. 2021;4(3):e210753. doi:10.1001/jamanetworkopen.2021.0753**

### Data
**Data available:** Yes
**Data types:** Deidentified participant data
**How to access data:** Harleen.K.Sandhu@uth.tmc.edu **When available:** With publication

### Supporting Documents
**Document types:** None

### Additional Information
**Who can access the data:** Researchers whose proposed use of the data has been approved
**Types of analyses:** Any reasonable purpose
**Mechanisms of data availability:** After approval of a proposal with a signed data access agreement
